# 2062. In Vivo Efficacy of CD388, a Novel Drug Fc-Conjugate (DFC), Against Seasonal Subtypes of Influenza in Prophylaxis in Immune Competent Mice, and in a Severe Immunodeficient (SCID) Mouse Model

**DOI:** 10.1093/ofid/ofad500.132

**Published:** 2023-11-27

**Authors:** James Levin, Simon Döhrmann, Jason N Cole, Karin Amundson, Allen Borchardt, Thomas P Brady, Thanh Lam, Joanne Fortier, Amanda Almaguer, Leslie W Tari

**Affiliations:** Cidara Therapeutics, San Diego, CA; Cidara Therapeutics, San Diego, CA; Cidara Therapeutics, San Diego, CA; Cidara Therapeutics, San Diego, CA; Cidara Therapeutics, San Diego, CA; Cidara Therapeutics, San Diego, CA; Cidara Therapeutics, San Diego, CA; Cidara Therapeutics, San Diego, CA; Cidara Therapeutics, San Diego, CA; Cidara Therapeutics, San Diego, CA

## Abstract

**Background:**

Influenza remains a significant public health concern due to the limited efficacy of vaccines. Cidara has developed CD388, a multivalent conjugate of a dimeric neuraminidase inhibitor with a proprietary hIgG1 Fc domain engineered for extended half-life, currently in clinical development. Here we describe the efficacy of CD388 against a panel of geographically and temporally diverse influenza A and B subtypes in mouse prophylactic models, and against H1N1 in a SCID model.

**Methods:**

Efficacy studies were conducted in BALB/c mice lethally challenged with influenza virus. CD388 was administered intramuscularly (IM), seven days prior to challenge. Animals were monitored daily for 21 days or longer for survival (< 20% BW loss). For SCID studies, mice were dosed with CD388 two hours prior to lethal challenge with influenza A H1N1. Oseltamivir and baloxavir were included as comparators.

**Results:**

Across all influenza subtypes, CD388 treatment at ≤1 mg/kg resulted in 100% survival (**Table 1**). Daily BW measurements of treatment groups supported survival findings and showed a transient BW loss (generally between 5 and 15% of starting weight), before animals recovered weight.

In SCID studies, the median time of death for vehicle treated animals was seven days. Treatment with oseltamivir (5 mg/kg, bid x5d) and baloxavir (15 mg/kg, bid x1d) at their human equivalent doses extended the median survival time to Day 10 and 16, respectively (**Figure 1**). In contrast, a single dose of CD388 at 1 mg/kg extended the median time of death to beyond Day 21. A dose response was evident at higher CD388 doses with the median time of death extended to Day 30 and 35 for 3 and 10 mg/kg groups, respectively.

**Conclusion:**

This data demonstrated a single IM administration of CD388 fully protected animals from lethal challenge against seasonal influenza A and B. Importantly, CD388 was found to be efficacious in a severe model of immune deficiency. This data supports the prophylactic use of CD388 to prevent seasonal influenza in individuals.

**Figure:**
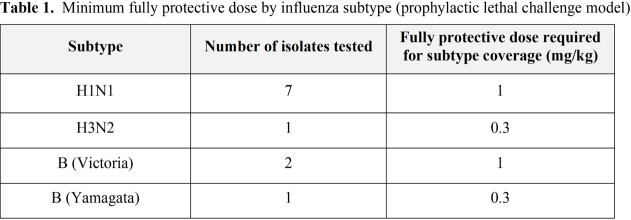

**Figure:**
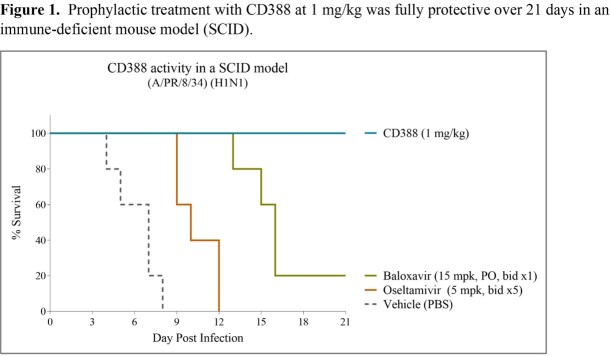

**Disclosures:**

**James Levin, PhD**, Cidara Therapeutics: Stocks/Bonds **Simon Döhrmann, PhD**, Cidara Therapeutics: Stocks/Bonds **Jason N. Cole, Ph.D.**, Cidara Therapeutics: Stocks/Bonds **Karin Amundson, BSc**, Cidara Therapeutics: Stocks/Bonds **Allen Borchardt, PhD**, Cidara Therapeutics: Stocks/Bonds **Thomas P. Brady, Ph.D.**, Cidara Therapeutics: Stocks/Bonds **Thanh Lam, PhD**, Cidara Therapeutics: Stocks/Bonds **Joanne Fortier, BSc**, Cidara Therapeutics: Stocks/Bonds **Amanda Almaguer, Bachelors**, Cidara Therapeutics: Stocks/Bonds **Leslie W. Tari, Ph.D.**, Cidara Therapeutics: Stocks/Bonds

